# Retraction: Bcl-2 Regulates HIF-1α Protein Stabilization in Hypoxic Melanoma Cells via the Molecular Chaperone HSP90

**DOI:** 10.1371/journal.pone.0268235

**Published:** 2022-05-03

**Authors:** The PLOS ONE Editors

After publication of this article [1], concerns were raised about the following results:

- Fig 1B: Similarities were noted between the β-actin panels for the "dense" and "4 days" experiments.
- Fig 1D: There appear to be vertical discontinuities in background between lanes 1/2 and between lanes 2/3 in the HIF1α 2 ml panel.
- Fig 1E: Similarities were noted between the Bcl2/5 and Bcl2/37 bands in the HIF-1α Insulin panel.
- Fig 2C: When aspect ratios are adjusted, the β-actin panels for puro and Bcl2/5 appear more similar than would be expected for different experimental results.
- Fig 3B: There is an irregularity after lane 1 of the left bcl-2 blot, suggestive of possible image splicing.
- Fig 6A: The β-actin data in lane 3 of the Bcl2/5 panel and in lane 1 of the Bcl2/37 panel appear similar.

The authors noted that the original primary data supporting Fig 1B, 1D and 1E, and several other figures in the article are no longer available. They provided data from replication experiments to support the Fig 1B and E results, but the replication data did not fully resolve the concerns about the published figures.

In light of the above issues, the *PLOS ONE* Editors retract this article.

DT, CB, and DDB agreed with retraction but stand by the article’s findings. EZ did not reply or could not be reached. The corresponding author noted that MD and GZ are deceased.
